# An adaptable method using human mixed tissue ratiometric controls for benchmarking performance on gene expression microarrays in clinical laboratories

**DOI:** 10.1186/1472-6750-11-38

**Published:** 2011-04-12

**Authors:** P Scott Pine, Barry A Rosenzweig, Karol L Thompson

**Affiliations:** 1 Center for Drug Evaluation and Research, US Food and Drug Administration, Silver Spring, MD 20993 USA; 2 National Institute of Standards and Technology, Gaithersburg, MD 20899 USA

## Abstract

**Background:**

Molecular biomarkers that are based on mRNA transcripts are being developed for the diagnosis and treatment of a number of diseases. DNA microarrays are one of the primary technologies being used to develop classifiers from gene expression data for clinically relevant outcomes. Microarray assays are highly multiplexed measures of comparative gene expression but have a limited dynamic range of measurement and show compression in fold change detection. To increase the clinical utility of microarrays, assay controls are needed that benchmark performance using metrics that are relevant to the analysis of genomic data generated with biological samples.

**Results:**

Ratiometric controls were prepared from commercial sources of high quality RNA from human tissues with distinctly different expression profiles and mixed in defined ratios. The samples were processed using six different target labeling protocols and replicate datasets were generated on high density gene expression microarrays. The area under the curve from receiver operating characteristic plots was calculated to measure diagnostic performance. The reliable region of the dynamic range was derived from log_2 _ratio deviation plots made for each dataset. Small but statistically significant differences in diagnostic performance were observed between standardized assays available from the array manufacturer and alternative methods for target generation. Assay performance using the reliable range of comparative measurement as a metric was improved by adjusting sample hybridization conditions for one commercial kit.

**Conclusions:**

Process improvement in microarray assay performance was demonstrated using samples prepared from commercially available materials and two metrics - diagnostic performance and the reliable range of measurement. These methods have advantages over approaches that use a limited set of external controls or correlations to reference sets, because they provide benchmark values that can be used by clinical laboratories to help optimize protocol conditions and laboratory proficiency with microarray assays.

## Background

As one of the first 'omic scale technologies in wide-spread use, microarrays provide a test case for addressing issues associated with highly multiplexed assays and high dimensional datasets. During the last decade, community-wide efforts were initiated to address concerns that arose with the increased use of microarray technology in research. These concerns include data comparability, the lack of universal controls for gene expression experiments, the need for data reporting standards, and methods for determining appropriate statistical approaches. The MicroArray Quality Control (MAQC) projects led by the FDA [1,2], the External RNA Controls Consortium under the leadership of the National Institute of Standards and Technology [3], and the Microarray Gene Expression Database (MGED) group which developed the MIAME (Minimum Information About a Microarray Experiment) standards [4] have laid the foundation for establishing confidence in microarray measurements, an essential step for the use of this technology in biomedical applications.

The use of microarray-based clinical tests, although currently limited, is expected to expand with the increasing incorporation of personalized medicine into medical practice. The availability of clinically directed standards would help promote the development of highly multiplexed gene expression assays in laboratory medicine [5]. The use of microarrays in clinical diagnostics will require assurance that clinical laboratories are and remain proficient in using this technology. Microarrays are designed for high throughput assessment of relative mRNA levels, but comparative expression measurements are constrained by limits in the dynamic range of detection [6]. Expression ratios derived from microarray data are compressed in comparison to quantitative real time polymerase chain reaction (qRT-PCR) assay data, especially for probes with high signal intensities [7]. The dynamic range and reliability of measurements on microarray assays can be further compromised by suboptimal laboratory proficiency with this technology. Routine performance assessments using reference samples are highly recommended as a best practice for the generation of high quality microarray data [8]. External RNA controls provided by array manufacturers have utility for evaluating the success or failure of certain sample processing steps but are limited in number and, because they do not span the range of intensities in biological samples, are not representative of all genes on a microarray [9]. The biologically complex samples used in the MAQC-I project to assess microarray data comparability across platforms have been widely used but, unlike samples in a typical analysis, are not closely related. MAQC-I sample A is a universal human reference RNA (UHRR) prepared by pooling RNA from multiple cell lines [10] and MAQC-I sample B consists of human brain reference RNA (HBRR) prepared from multiple individuals [1]. The difference in mRNA content between MAQC-1 samples A and B hinders the use of MAQC sample titrations to measure the ability to detect known changes between samples [11]. Typically, the MAQC samples have been used for performance comparisons by correlation of results to the reference microarray datasets or to the qRT-PCR data generated for a subset of analytes in the MAQC samples on the TaqMan platform (for examples, see [12,13]). Correlations to historical reference sets measure the degree of similarity to benchmark data but not necessarily an improvement in performance over reference set levels.

Objective criteria to measure performance on microarrays can be developed for samples which are designed to have known differences in expression. We developed a system for assessing technical performance with microarray assays that involves two biologically complex samples (mixed tissue ratiometric controls (MTRC)) that are representative of experimental samples [14]. MTRC are two samples with three or four RNA components mixed in different proportions. Technical performance is based on quantifiable measures of diagnostic accuracy and the reliable region of measurement. Diagnostic accuracy is the ability of a microarray assay to correctly detect true positive and negative changes in the MTRC samples based on differences in tissue-selective transcripts that are consistent with the ratios of mixed tissue RNA. The area under the curve (AUC) from receiver operating characteristic (ROC) plots summarizes the technical performance in a single benchmark value based on measurements of over 100 true positive and negative analytes in the MTRC. The utility of this approach has been evaluated using rat whole genome microarrays with mixtures of total RNA from four rat tissues (liver, brain, testis, and kidney) known to have distinctly different expression patterns [14,15]. Translating this approach for laboratories that analyze clinical samples is challenged by quality issues with human tissue RNA. In this manuscript, we describe a method for the design and use of performance standards for human gene expression arrays that use commercially available RNA and include metrics that identify the most reliable region of the dynamic range of measurement.

## Results

### Selection of components for the human MTRC

Mixed tissue ratiometric controls for monitoring performance with microarrays are two samples that are composed of RNA of high and reproducible quality from 3 or 4 different tissues that are sufficiently different in gene expression. The samples are designated MTRC-3 or MTRC-4 if they are composed of three or four different RNA sources, respectively. The four components of the human MTRC-4 used as the example in this study are UHRR, HBRR, liver RNA, and skeletal muscle RNA. Two components of the MTRC-4 (UHRR and HBRR) were chosen because they have been extensively tested in the MAQC-I project and are commercially available in large lots. The additional components of the MTRC-4 were chosen from the normal human tissues that had high levels of selective gene expression in published reports. Son *et al. *identified testis, liver, brain (cerebellum and cerebrum), skeletal muscle, and heart as the tissues with the highest number of organ-specific genes using a set of cDNA microarray data from 19 different organs from 30 individual donors [16]. Of the 46 human tissue samples and cell lines analyzed on Affymetrix Human Genome U95A arrays by Su *et al.*, the largest numbers of specifically expressed gene transcripts were found in testis, pancreas, liver, placenta, thymus, salivary gland, kidney, heart, and cerebellum [17]. We surveyed the quality of commercial sources of RNA for several of the tissues with high levels of selective expression (see Table 1). Of the six tissue sources tested in addition to HBRR and UHRR, RNA from human liver and skeletal muscle had the highest level of sample integrity across multiple lots. All of the lots of human liver and skeletal muscle RNA surveyed had RNA Integrity Number (RIN) values higher than or equal to 8, which is the threshold level of RNA quality that had no effect on the detection of known changes in gene expression on Affymetrix expression arrays [18].

**Table 1:** Source and quality of human tissue RNA used in testing

						MTRC Batch				
						
Tissue	Provider	Catalogue No.	**Lot No**.	RIN	Analyte Selection	1	2	3	4	5
			120000320	9.4				X		
Liver	Ambion	AM7960	40000129	9.1		X	X			
			811001	8.2	X				X	
			905002	8.1						X

	CHTN^a^	NA	NA	8.7		X				
Skeletal Muscle										
			811001	8.5	X			X	X	
	Ambion	(AM7982)	2060298	8.3			X			
			906002	8	X					X

HBRR	Ambion	AM6050	105055201A	7.6	X	X	X	X	X	X

UHRR	Stratagene	740000	1130623	8.2	X	X	X	X	X	

			580049	8.2						
Lung	Stratagene	540019	6051745	8.2						
			6037468	6.2						

Kidney	Ambion	AM7976	70100119	7.8						

Testes	Ambion	AM7972	5060396	7						

Placenta	Ambion	AM7950	202009	6.7						

^a^Cooperative Human Tissue Network

### Selection of analytes for the human MTRC-4

Performance assessments made using the MTRC are based on tissue-selective analytes. For MTRC analyte selection, tissue-selectivity was defined as an average signal intensity that is ten-fold higher in one tissue than the other tissues in the MTRC. For the MTRC-4, UHRR, HBRR, liver RNA, and skeletal muscle RNA were individually labeled and hybridized to Affymetrix Human Genome U133A 2.0 arrays on separate dates to create three replicate datasets. For each probe set in each tissue, the difference between its log_2 _signal intensity in that tissue and its maximum value in the remaining tissues was calculated to determine a tissue-selective index (TSI) for each dataset. Using a mean TSI cutoff of 3.22 log_2 _units, a total of 1035 tissue-selective analytes were identified for the MTRC-4 that included 429 HBRR-, 255 liver RNA-, 197 UHRR-, and 154 skeletal muscle RNA-selective probe sets (see Additional file 1 - Lists of MTRC-4 analytes for Affymetrix HG-U133A 2.0 arrays).

### Evaluation of components for the human MTRC-4

UHRR, liver RNA, HBRR, and skeletal muscle RNA were mixed together in two samples to yield ratios of 1:1, 1.5:1, 2:1, and 1:4, respectively, between Mix1 and Mix2 in the MTRC-4 tested in this study. Commercial sources of human liver and skeletal muscle RNA were available in lots prepared from single individuals in limited amounts. To assess the impact of the use of different lots of RNA on the reproducibility of results with the MTRC-4, we compared 6 single assays of the MTRC-4 that all used Method 2A for target preparation but different lots of liver RNA and of skeletal muscle RNA (Batches 1-4 in Table 1). An AUC was derived for each singlicate assay using the log_2 _ratio method [14] and used for the comparisons. There was a statistically significant difference in AUC using the MTRC-4 made with skeletal muscle RNA prepared from donor tissue (Trial 1) compared to the MTRC-4 made with commercial sources of skeletal muscle RNA (Table 2). The AUCs for 1.5-fold and 2-fold change detections were statistically different between Trial 1 using MTRC-4 Batch 1 and the five datasets that used MTRC-4 Batches 2-4. The 4-fold AUC was significantly different between Trial 1 and two of the replicate datasets of MTRC-4 Batch 4 (P < 0.05). Batches 2, 3 and 4 all used commercial sources of RNA. Different lots of liver and skeletal muscle RNA were used in Batch 2 than in Batches 3 and 4. Batches 3 and 4 share the same lot of skeletal muscle RNA but contain different lots of liver RNA (see Table 1). No difference in 2-fold or 4-fold AUC was observed between Batches 2, 3, and 4. The 1.5-fold AUC was significantly different between Trial 2 using Batch 2 RNA and datasets using Batch 3 or 4 RNA (P < 0.05). These results indicate that the human MTRC-4 could be generated using different commercial lots of good quality human RNA with minor differences in performance. Although these differences were less than those observed between technical replicates with some of the methods used in this study (data not shown), the same batch of MTRC should be used for systematic assessments of the effect of specific variables on laboratory performance.

**Table 2:** Differences in performance between MTRC-4 batches labeled with the same method

		AUC		
		
Dataset	Batch	4-fold	2-fold	1.5-fold
Trial 1	1	0.990*	0.991**	0.944**
Trial 2	2	0.999	1.000	0.963*
Trial 3	3	0.995	1.000	*0.981*
2A-4_rep1	4	*1.000*	1.000	*0.982*
2A-4_rep2	4	0.999	1.000	*0.983*
2A-4_rep3	4	*1.000*	1.000	*0.983*

*Statistically different from italicized values in the same column (P < 0.05).

**Statistically different from all other values within the same column (P < 0.05).

### Evaluation of diagnostic performance

To demonstrate the utility of the human MTRC for assessing small improvements in performance, we evaluated the impact of differences in labeling protocols and kits on benchmark values. Target labeling methods are a recognized source of variation in microarray data [19,20]. Minor differences in target labeling methods can introduce systematic bias in signal intensity, due to differences in target sequence or length, which may impact the statistical detection of differential gene expression. For this study, Batch 4 of the MTRC-4 was labeled using different commercially available kits with several variations in laboratory protocol that were within the manufacturers' recommendations for length of time of labeling reaction, amount of total RNA input, and amount of target hybridized to arrays (see Table 3). The studies included two generations of the array manufacturer's labeling kit (Affymetrix IVT and IVT Express) that use the Eberwine method to generate linear amplified RNA (aRNA) targets and one kit that generates a single stranded (ss)-cDNA target through isothermal linear amplification of cDNA (Nugen Ovation V2) [21].

**Table 3:** Comparison of AUCs derived with the MTRC-4 and MTRC-3 using different labeling protocols

				Variations in method				AUC		
						
Dataset	Method	MTRC design^a^	Kit	Total RNA input (μg)	Labeling time (hr)	Target hybridized (μg)^b^		4-fold	2-fold	1.5-fold
1A-4	1A	4	IVT Express	0.1	16	6.5		0.99	0.99	*0.95*
1B-4	1B	4	IVT Express	0.1-0.2	4	6.5		0.99	0.98	*0.93*
2A-4	2A	4	IVT	5	16	6.5		0.98	0.97	*0.93*
2A-3	2A	3	IVT	5	16	6.5		-	-	*0.95*
3A-4	3A	4	Ovation	0.02	1	2.2		0.99	0.97	0.87*
3B-4	3B	4	Ovation	0.02	1	0.55		0.99	0.98	0.89*
3C-3	3C	3	Ovation	0.1	1	0.55		-	-	0.88*

^a^The MTRC-4 design was tested with MTRC Batch 4. The MTRC-3 design was tested with MTRC Batch 5.

^b^The target is aRNA for Methods 1 and 2 and cDNA for Method 3.

*Statistically different from italicized values (P < 0.01).

For each labeling method, a p-value was calculated for each MTRC-4 analyte using a paired t-test comparison of normalized log_2 _signals from array data for three replicate assays of Mix1 and Mix2. A ROC-plot was generated for each of the true positive subsets in the MTRC-4 datasets (for 1.5-, 2-, and 4-fold changes) compared to the true negative (1-to-1) subset and the corresponding AUCs calculated as described previously [14]. No significant difference in the accurate detection of changes of two-fold or greater was observed between labeling methods (Table 3). However, there was a statistically significant difference in 1.5-fold AUC between datasets generated with aRNA targets and datasets generated with ss-cDNA targets (P < 0.01). Of the ratios in the MTRC-4, 1.5-fold changes are more sensitive to noise [14] which can be contributed by factors such as variation between replicates. A lower degree of reproducibility between analyte ratios for technical replicates was observed for data generated using Method 3 compared to Method 1, which may be due in part to the greater familiarity of our laboratory with Methods 1 and 2. A Spearman's rank correlation coefficient (ρ) between each possible comparison of technical replicates was calculated using the log_2 _ratios of 1035 tissue-selective analytes. The mean ρ (± standard deviation) for datasets 1A-4, 1B-4, 3A-4, and 3B-4 was 0.897 ± 0.02, 0.928 ± 0.004, 0.861 ± 0.019, and 0.852 ± 0.003, respectively. No significant difference in 1.5-fold AUC was found between two versions of a manufacturer's kit (datasets 1A-4 and 2A-4) or between variations in the aRNA protocol in the time of labeling (datasets 1A-4 and 1B-4). There was also no significant difference in the AUCs when the amount of ss-cDNA hybridized to arrays was changed within a protocol (dataset 3A-4 vs. 3B-4).

### Determination of reliable range of measurement

**Figure 1:**
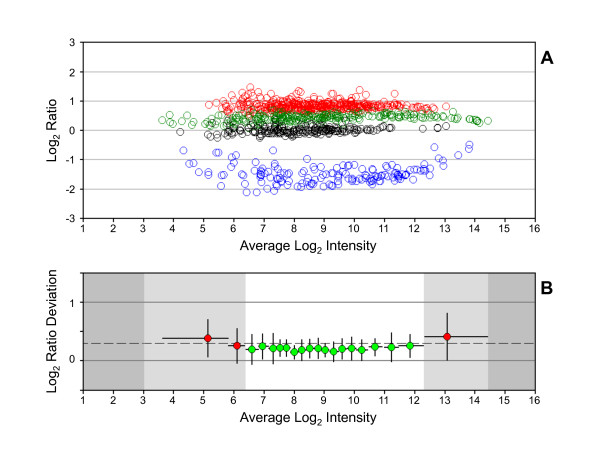
**Log_2 _ratio distribution for MTRC dataset 1A-4**. (A) RI-plot. Each analyte is represented by an open circle that is colored for tissue selective expression. Red = HBRR-selective (target log_2 _ratio of 1), green = liver RNA-selective (target log_2 _ratio of 0.585), blue = skeletal muscle RNA-selective (target log_2 _ratio of -2), and black = UHRR-selective (target log_2 _ratio of 0). (B) LRD-plot. Each circle corresponds to the mean LRD for analytes binned by mean log_2 _intensity and is colored by statistical test result. Green = passed χ^2 ^test and red = failed χ^2 ^test. y-error bars = *s *per bin and x-error bars = intensity range of bin. White region = reliable range and dark gray region = outside the dynamic range. Dashed line = *s *(the standard deviation of the log_2 _ratios for all probe sets).

Comparative expression data from microarrays is constrained at the upper and lower regions of signal intensity by the output range of the scanner and the noise inherent in measuring low intensity signals, respectively [6]. A Ratio-Intensity plot (RI-plot) of MTRC data provides a visualization of the intensity-dependent separation between analytes with different target ratios, as shown in Figure 1A for dataset 1A-4. The regions at the upper and lower limits of detection where data converge and the region within the dynamic range that remains linear can be established with further analysis. We developed a metric, the reliable range, to quantify the linear region of the dynamic range of measurement using the MTRC analytes. This metric can also serve as a benchmark value for process improvement. A reliable range of measurement can be calculated by plotting the log_2 _ratio deviation (LRD) for MTRC analytes as a function of signal intensity. The LRD is the difference between the observed and expected ratio for each MTRC analyte. In LRD-plots, analytes are rank ordered by intensity and divided into bins containing approximately equal numbers of data points. Next, the mean and standard deviation of the LRD and the mean and range of intensities are calculated for each bin and plotted. Finally, the LRD is used in a chi-square test to evaluate each bin as follows:(1)

where *n *is the number of analytes per bin. The standard deviation (*s*) in the log_2 _ratios of all probe sets is used as the variance estimate and a comparison to the χ^2 ^distribution at α = 0.01 is used to evaluate the goodness of fit within each bin. The set of contiguous bins that pass the χ^2 ^test is identified and used to define the upper and lower limits of a reliable range of measurement using the maximum and minimum intensities from the "passing" bins, respectively (see Figure 1B). By binning LRD values by signal intensity, the LRD-plot demarcates regions of the dynamic range where ratios significantly deviate from their target values due to noise or ratio compression.

**Figure 2:**
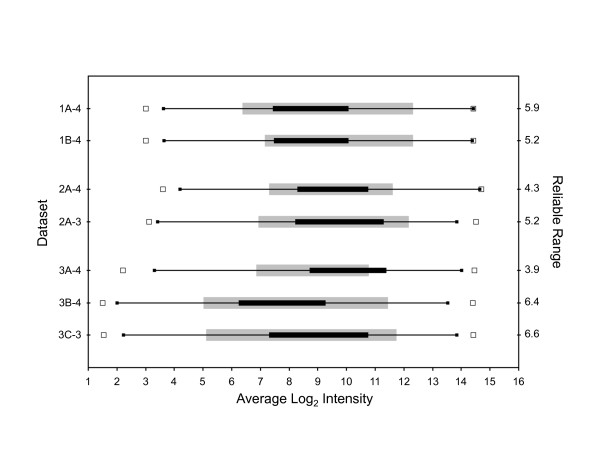
**Reliable range and analyte distributions for MTRC-4 and MTRC-3 datasets that used different labeling protocols**. The distribution of MTRC analytes in each dataset is depicted as a boxplot with the interquartile range represented by a black rectangle, connected by whiskers to the minimum and maximum signal for all analytes (solid boxes). Open squares correspond to the ends of the dynamic range, the minimum and maximum signals for all probe sets. Gray rectangles represent the reliable range for each labeling method. The length of the reliable range in log_2 _units is indicated on the right y-axis.

**Figure 3:**
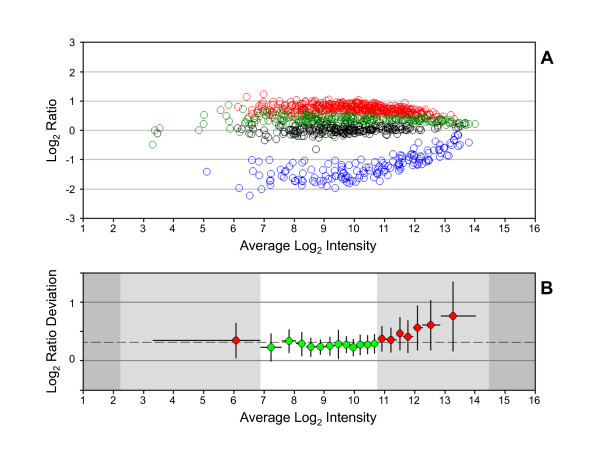
**Log_2 _ratio distribution for MTRC dataset 3A-4**. (A) RI-plot. Same format as for Figure 1. (B) LRD-plot. Same format as for Figure 1.

**Figure 4:**
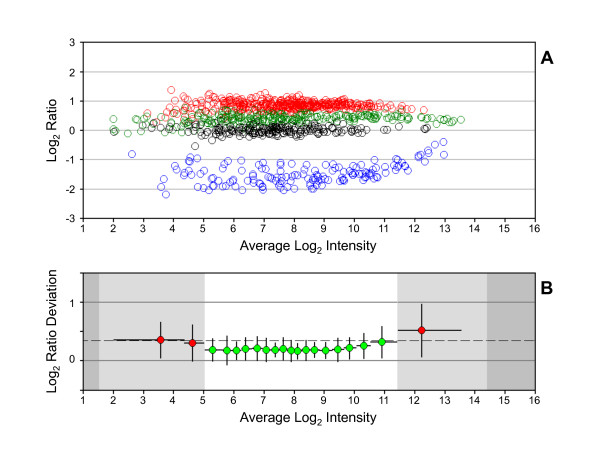
**Log_2 _ratio distribution for MTRC dataset 3B-4**. (A) RI-plot. Same format as for Figure 1. (B) LRD-plot. Same format as for Figure 1.

The reliable range was calculated for each dataset that was generated using a different labeling method (Figure 2). The shortest reliable range was observed for dataset 3A-4. The LRD-plot for dataset 3A-4 shows that about 40% of the MTRC analytes lay outside the reliable range (Figure 3A-B). A similar result is seen in Figure 2, where the interquartile range of the MTRC analytes for dataset 3A-4 extends beyond the reliable range. An adjustment in the 3A-4 protocol was made in Method 3B that reduced the amount of target hybridized to arrays and resulted in a marked increase in length of the reliable region of measurement (Figures 2 and 4A-B). About 85% of the MTRC-4 analytes reside within the reliable range in dataset 3B-4. After protocol optimization, the results are in agreement with previous studies [22] that showed a larger dynamic range of measurement with the linear isothermal amplification methods compared to T7 RNA polymerase based methods traditionally used for sample generation on Affymetrix arrays.

### Optional design for the human MTRC

**Figure 5:**
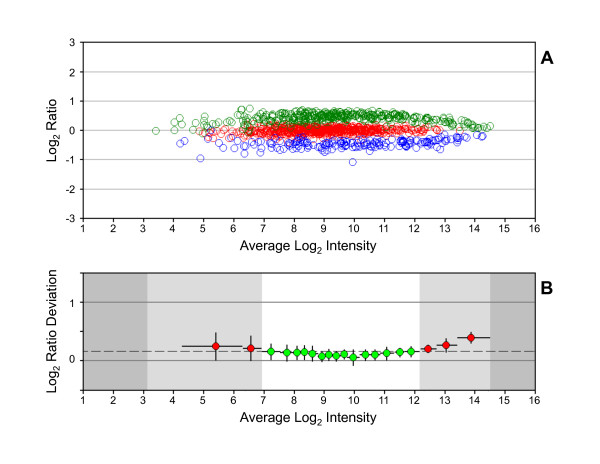
**Log_2 _ratio distribution for MTRC dataset 2A-3**. (A) RI-plot. Each analyte is represented by an open circle that is colored for tissue selective expression. Green = liver RNA-selective (target log_2 _ratio of 0.585), blue = skeletal muscle RNA-selective (target log_2 _ratio of -0.585), and red = HBRR-selective (target log_2 _ratio of 0). (B) LRD-plot. Same format as for Figure 1.

Mixed tissue ratiometric controls can also be designed that focus on a single target ratio of interest. We examined the utility of three component MTRC (MTRC-3) designed to have one component that is 1.5-fold higher in Mix1 than Mix2, one component that is 1.5-fold lower in Mix1 than Mix2, and one component at a 1-to-1 ratio for normalization. To choose which RNA source in the MTRC-4 to exclude in the MTRC-3, we systematically omitted one of the four RNAs in the MTRC-4 and recalculated the number of tissue-selective analytes amongst the remaining three RNAs in the comparison. Excluding skeletal muscle RNA, liver RNA, or HBRR produced 1085, 952, or 773 analytes, respectively. The highest number of tissue-selective analytes (1151) was achieved by leaving out UHRR (see Additional file 2 - Lists of MTRC-3 analytes for Affymetrix HG-U133A 2.0 arrays). These results were expected, since the UHRR is designed to cover a large portion of the transcriptome [10]. Further evaluation of the use of the MTRC-3 design in performance assessments was carried out with mixes that contained human skeletal muscle RNA, human liver RNA, and HBRR in 1:1.5, 1.5:1, and 1:1 ratios. An example of an RI-plot and an LRD-plot generated using the MTRC-3 is shown in Figure 5.

Diagnostic performance measured with the 1.5-fold AUC was similar between MTRC-3 and MTRC-4 datasets that were generated using the same labeling methods. No significant difference was observed between 1.5-fold AUCs for MTRC-3 dataset 2A-3 and MTRC-4 dataset 2A-4 that were both labeled using Method 2A or between MTRC-3 dataset 3C-3 and MTRC-4 datasets 3A-4 and 3B-4 that all used variations of Method 3, despite differences in the identity and number of analytes between the two MTRC designs (Table 3). Similar to the MTRC-4, significant differences were observed between MTRC-3 datasets generated with different types of targets (aRNA vs. ss-cDNA). A statistically significant difference in diagnostic performance was observed between dataset 2A-3 that used the MTRC-3 with a linear isothermal amplification method and dataset 3C-3 that used the MTRC-3 with the T7 RNA polymerase based amplification method to generate target for hybridization to arrays (Table 3). A longer reliable range and dynamic range of measurement seen for Method 3 compared to Methods 1 and 2 with the MTRC-4 and in published reports [22] were also observed with MTRC-3 dataset 3C-3 compared to MTRC-3 dataset 2A-3 (Figure 5).

## Discussion

The use of human MTRC samples which contain known changes in expression along the dynamic range of measurement with the reliable range metric introduced in this manuscript provide a mechanism for clinical laboratories conducting microarray assays to benchmark performance, assess laboratory proficiency, and measure process improvement. Process drift or process improvement, introduced through automation of procedures or changes in reagents, equipment, operator, or platforms, may involve relatively subtle effects on microarray data. For example, in this study we evaluated the effect of a protocol option with a reduced time for target labeling (Method 1A vs. 1B). Our results indicated that the shorter incubation time could be used without significantly impacting assay performance. For these process improvement applications, we have found that a test for accurate measurement of 1.5-fold changes in comparative expression is a better discriminator of subtle effects on performance than assays for changes that are two-fold or higher. A target ratio of 1.5-fold is included in the MTRC-4 as one of 4 components or in the MTRC-3 as the single true positive change. We recommend using the 1.5-AUC metric, which incorporates a measure of diagnostic accuracy and proficiency in replication of results with the MTRC, as a first tier test for process improvement.

Although correlation to reference sets or within replicates is often used to assess performance on microarrays, this metric is of limited value for measuring improvement in laboratory techniques. Datasets generated using methods 3A and 3B had similar mean Pearson's correlation coefficients (data not shown) and Spearman's rank correlation coefficients for ratio reproducibility between technical replicates. However, a clear improvement in the RI-plot (Figures 3A and 4A) and in the reliable range value (Figure 2) could be observed with a change in protocol that reduced the amount of target hybridized to arrays (Method 3B vs. 3A). Optimal results with microarray assays are best obtained using measurements that avoid the boundaries of the dynamic range [6]. The MTRC contain hundreds of analytes with known target ratios that span the dynamic range of measurement and that serve as input values into reliable range estimates. The reliable range of measurement that is calculated with the MTRC from LRD-plots is a benchmark value for evaluating an acceptable performance level for laboratory protocols.

We demonstrated two options in sample design for mixed tissue ratiometric controls. The same metrics (ROC-plot AUCs, reliable range from LRD-plots) can be calculated with either design and yielded similar results. The MTRC-3 design is easily tunable to measure a target ratio of interest and has an advantage over the MTRC-4 in requiring only 3 different sources of reliably good quality human tissue RNA. The human MTRC-4 is directly comparable to the previously described rat MTRRM design used to illustrate the utility of a ROC-plot AUC to identify outliers in performance in a large set of proficiency testing data [14,15].

Previously, we have described methodology for using the MAQC samples A and B, and two dilutions of A and B, for performance assessments based on ROC-plot metrics [23]. However, these samples have limitations. The MAQC samples A-D each contain 1 or 2 tissues and the observed differences between total RNA components do not correspond to mixed ratios without adjustment for the projected difference in mRNA content between UHRR and HBRR [11]. The MTRC-4 design tested in this study limits the skewing effect of UHRR on observed ratios by using it in a 1:1 ratio. The qRT-PCR data generated for the MAQC-I project can be used as the basis for selecting limited sets of true positive and true negative analytes [23]. However, this approach yields a low number of analytes with limited statistical power for calculating an AUC from a ROC-plot or determining the reliable range from an LRD-plot. The addition of 1 or 2 different human tissue RNA components to MAQC samples A and/or B creates a two-sample system that is better suited for monitoring process improvement with microarray assays. Although this study is limited to Affymetrix gene expression arrays, we have previously shown that the MTRC samples and metrics can be used on other platforms [14,15].

## Conclusions

While DNA microarrays are widely used in medical research in developing improved assays for prediction or diagnosis of disease or for assessing the efficacy and adverse effects of pharmaceuticals, their use in clinical practice is currently limited. One microarray-based transcriptional profiling assay is currently approved by the FDA for predicting responsiveness to certain therapeutic interventions for breast cancer and several others are under development [24]. The expanded use of microarray technology in clinical medicine would be enabled by the implementation of methods for monitoring and optimizing laboratory performance. The methods for measuring performance on human genome-wide microarrays that are described in this manuscript fit this purpose and can be performed by clinical laboratories using samples prepared from commercially available sources.

## Methods

### RNA

Human tissue RNA was obtained from Ambion (Applied Biosystems, Carlsbad, CA) or Stratagene (Agilent Technologies Inc., Santa Clara, CA). The catalog and lot numbers of the products tested are indicated in Table 1. RNA was also prepared from human skeletal muscle tissue using a Qiagen TissueLyser and Qiagen RNeasy miniprep kits (Qiagen, Valencia, CA). The donor tissue was obtained from amputation procedures through the Cooperative Human Tissue Network which is funded by the National Cancer Institute. The protocol for this study was reviewed by the FDA Research Involving Human Subjects Committee.

RNA quantification and assessment of purity was performed on a NanoDrop spectrophotometer (ThermoScientific, Wilmington, DE). RNA quality was assessed using an Agilent RNA 6000 Nano kit, an Agilent Bioanalyzer (Agilent Technologies, Inc.), and the manufacturer's software to assign RINs [25]. The RINs that were measured for different lots of commercial human RNA are in Table 1.

### Analyte Selection

UHRR, HBRR, liver RNA, and skeletal muscle RNA were individually labeled using the 3' IVT Express kit (Method 1A) and hybridized to Human Genome U133A 2.0 arrays (Affymetrix, Santa Clara, CA). Three replicate datasets were created by labeling and hybridizing the four tissue RNA samples on separate dates to introduce technical variation. The commercial lots of RNA used for analyte selection are indicated in Table 1. For each replicate dataset, the log_2 _signal intensities were derived and quantile normalized using the Robust Multichip Analysis (RMA) algorithm in the Affymetrix Expression Console software. A mean TSI for each probe set was calculated from the three replicate datasets and the threshold for tissue selectivity was a mean TSI greater than 3.22 log_2 _units. For the MTRC-4, 429 HBRR-, 255 liver RNA-, 197 UHRR-, and 154 skeletal muscle RNA-selective analytes were identified (see Additional file 1 - Lists of MTRC-4 analytes for Affymetrix HG-U133A 2.0 arrays). With omission of the UHRR from the comparison, 586 HBRR-, 384 liver RNA-, and 181 skeletal muscle RNA-selective analytes were identified as analytes for the MTRC-3 (see Additional file 2 - Lists of MTRC-3 analytes for Affymetrix HG-U133A 2.0 arrays).

### Mixture Design

Four batches of MTRC-4 were prepared using total RNA from four human tissues from different lots (Table 1). Batches 1-3 were tested in singlicate measurements (Trials 1-3) and Batch 4 was used in all of the other MTRC-4 datasets. A 100 μg batch of MTRC-4 Mix1 contains 20 μg UHRR, 30 μg liver RNA, 40 μg HBRR RNA, and 10 μg skeletal muscle RNA. A 100 μg batch of MTRC-4 Mix2 contains 20 μg UHRR, 20 μg liver RNA, 20 μg HBRR RNA, and 40 μg skeletal muscle RNA. The ratio of total RNA contributed by UHRR, liver, HBRR and skeletal muscle between Mix1 and Mix2 in the MTRC-4 is 1:1, 1.5:1, 2:1, and 1:4, respectively. UHRR was used for the 1-to-1 component in the MTRC-4 to minimize the impact on the observed mixed ratio due to the higher fraction of mRNA in the cell line derived total RNA than in sources of tissue RNA like the HBRR [11]. Human liver RNA, which was found to have a higher RIN value across commercial lots than the other components, was used for the ratio more sensitive to performance differences (1.5-fold). Of the remaining two components, the HBRR was selected for measuring 2-fold changes because it contained the larger number of tissue-selective analytes.

MTRC-3 were prepared from total RNA from three human tissues using the lots indicated for Batch 5 in Table 1 and mixed to provide a 1:1 ratio for HBRR and reciprocal 1.5:1 ratios for liver and muscle RNAs. A 100 μg batch of MTRC-3 Mix1 contained 25 μg HBRR RNA, 45 μg liver RNA, and 30 μg skeletal muscle RNA. A 100 μg batch of MTRC-3 Mix2 contained 25 μg HBRR RNA, 30 μg liver RNA, and 45 μg skeletal muscle RNA. With the HBRR as the 1-to-1 component in the MTRC-3, a similar number of true negatives (586 brain RNA-selective analytes) and true positives (565 combined liver RNA- and skeletal muscle RNA-selective analytes) could be used in the ROC-plot analyses.

### Target preparation

Labeled target was prepared from total RNA samples using one of the following three reagent kits: (1) 3' IVT Express kit (Affymetrix Part No. 901229), (2) IVT kit (Affymetrix Part No. 900449), or (3) Ovation RNA Amplification System V2 and the FL-Ovation cDNA Biotin module kits (Nugen Catalog Nos. 3100 and 4200). Labeled target was hybridized to Affymetrix Human Genome U133A 2.0 arrays in a GeneChip Hybridization Oven 640. The arrays were washed on an Affymetrix GeneChip Fluidics Station 450 using fluidics protocol FS450-002 and scanned using an Affymetrix GeneChip Scanner 3000 7G. The variations made in protocols for each target labeling method are listed in Table 3. For each method, a pair of MTRC was run in triplicate but processed on different days to create sets of technical replicates. The microarray data from this study are available in the ArrayExpress Archive at the European Bioinformatics Institute through accession number E-TABM-1091 http://www.ebi.ac.uk/arrayexpress.

### Normalization

Each dataset that was created using a different target labeling protocol or different batch of MTRC was processed separately with RMA. The selective analytes for the 1-to-1 component (UHRR for the MTRC-4 and HBRR for the MTRC-3) were used to normalize Mix2 with respect to Mix1. For each technical replicate, the difference in the 10% trimmed mean intensity between the Mix1 and Mix2 data for analytes in the 1-to-1 component was calculated and used to correct the Mix2 signal data.

### ROC-plots

For ROC-plot calculations using the MTRC, the true negatives are the tissue selective analytes for tissue RNA that is mixed in a 1-to-1 ratio between Mix1 and Mix2. Separate ROC-plots are generated for each true positive subset in the MTRC. For MTRC-4, the true positives are the 1.5-, 2-, and 4-fold changes and for the MTRC-3, the true positives are the 1.5-fold changes in both directions. For singlicate assays, analytes are ranked by log_2 _ratio [14]. For replicate assays, analytes are ranked by p-values calculated using a paired t-test comparison of the three Mix1 and Mix2 signals. AUCs were calculated using the trapezoidal method. Statistical analyses of differences in AUCs were performed as previously described [14] using the method of Hanley and McNeil [26].

### RI-plots

For each dataset, the normalized log_2 _signals were averaged across technical replicates of either Mix1 or Mix2. The average log_2 _signals () for Mix1 and Mix2 were then used to calculate the *ratio *(R) and *intensity *(I) for each analyte as follows:(2)(3)

### LRD-plots

For MTRC-4 datasets, the analytes (excluding the 1-to-1 component) were distributed into 19 bins of 42, with a single bin of 40 at the lowest intensity. For MTRC-3 datasets, the analytes (excluding the 1-to-1 component) were distributed into 20 bins of 28 analytes, omitting the 5 lowest intensity analytes. The standard deviation (*s*) ranged from 0.30 - 0.34 for MTRC-4 datasets and from 0.16 - 0.21 for MTRC-3 datasets.

## Competing interests

The authors declare that they have no competing interests.

## Authors' contributions

PSP performed the data analysis and developed the metrics. BAR prepared the MTRC and performed the RNA analysis and microarray assays. The concept and scope of the project were designed by KLT. PSP and KLT prepared the manuscript. All authors read and approved the final manuscript.

## Supplementary Material

Additional file 1 **Lists of MTRC-4 analytes for Affymetrix HG-U133A 2.0 arrays**. Analytes selective for HBRR, human liver RNA, human skeletal muscle RNA, and UHRR are identified by Affymetrix probe set identifier, gene name (annotation date 3-17-2008), and Tissue Selective Index (TSI). This file is formatted as an Excel spreadsheet. Click here for file

Additional file 2 **Lists of MTRC-3 analytes for Affymetrix HG-U133A 2.0 arrays**. Analytes selective for HBRR, human liver RNA, and human skeletal muscle RNA are identified by Affymetrix probe set identifier, gene name (annotation date 3-17-2008), and Tissue Selective Index (TSI). This file is formatted as an Excel spreadsheet. Click here for file
